# Patient and Caregiver Experiences With and Perceptions of Risk Evaluation and Mitigation Strategy Programs With Elements to Assure Safe Use

**DOI:** 10.1001/jamanetworkopen.2021.44386

**Published:** 2022-01-20

**Authors:** Ameet Sarpatwari, Beatrice L. Brown, Sarah A. McGraw, Sara Z. Dejene, Abdurrahman Abdurrob, Adrian J. Santiago Ortiz, Aaron S. Kesselheim

**Affiliations:** 1 Program On Regulation, Therapeutics, And Law (PORTAL), Division of Pharmacoepidemiology and Pharmacoeconomics, Department of Medicine, Brigham and Women’s Hospital and Harvard Medical School, Boston, Massachusetts; 2 The Hastings Center, Garrison, New York

## Abstract

**Importance:**

The US Food and Drug Administration (FDA) Amendments Act of 2007 authorized the FDA to impose safety requirements on drugs with important risks, such as prescriber certification or routine laboratory testing, to ensure that the benefits of use outweighed the risks. However, little is known about patient and caregiver experiences with these Risk Evaluation and Mitigation Strategy (REMS) programs with Elements to Assure Safe Use (ETASU).

**Objective:**

To understand patient and caregiver experiences with and perceptions of REMS programs with ETASU.

**Design, Setting, and Participants:**

This qualitative study included semistructured qualitative phone interviews conducted between 2016 and 2017, with initial analysis performed in 2017 and reanalysis performed in 2021. Adult patients prescribed natalizumab or sodium oxybate, adult patients or caregivers of adult patients prescribed vigabatrin, and adult female patients of reproductive age prescribed riociguat were included.

**Main Outcomes and Measures:**

Assessment of knowledge, decision-making, medication access, and perceptions of medical privacy.

**Results:**

Among 63 participants, 46 (73%) were female. Twenty-five participants (40%) had taken natalizumab, 10 (16%) riociguat, 15 (24%) sodium oxybate, and 10 (16%) vigabatrin. One participant had taken both natalizumab and vigabatrin; 4 (6%) were caregivers of patients using vigabatrin. Most participants expressed knowledge of REMS program requirements, but many lacked the insight that these requirements were part of an FDA-mandated special safety program and expressed difficulty understanding program education materials. REMS requirements made some participants more likely to initiate treatment. However, many reported burdens accessing medication, including the need to travel to certified prescribers or pharmacies. Manufacturer access to personal health information was also controversial, although some participants expressed an altruistic desire to assist others.

**Conclusions and Relevance:**

This qualitative study found that REMS programs with ETASU reassured patients and their caregivers about drug safety and helped support medication initiation. However, steps are needed to improve the quality of REMS educational materials, promote efficient medication access, and protect patient privacy.

## Key Points

**Question:**

How do patients and caregivers describe their experiences with Risk Evaluation and Mitigation Strategy (REMS) programs with Elements to Assure Safe Use (ETASU) mandated by the US Food and Drug Administration?

**Findings:**

In this qualitative study of 63 participants, some patients and caregivers of patients who received 1 of 4 drugs subject to a REMS program with ETASU—natalizumab, riociguat, sodium oxybate, and vigabatrin—reported being reassured by the program. However, many patients and their caregivers also reported difficulty understanding program educational materials, burdens accessing medications, and data-sharing concerns.

**Meaning:**

The findings of this study suggest that improving communication, access-related burdens, and privacy protections can help strengthen REMS programs with ETASU.

## Teaser

This qualitative study examines patient and caregiver experiences with and perceptions of Risk Evaluation and Mitigation Strategy programs with Elements to Assure Safe Use.

## Introduction

Congress passed the US Food and Drug Administration (FDA) Amendments Act of 2007 to improve prescription drug safety regulation in the wake of a series of widely reported cases in which FDA-approved drugs were found to cause deadly adverse events.^1^ One key provision of the Act granted the FDA the power to require that manufacturers implement Risk Evaluation and Mitigation Strategy (REMS) programs for certain drugs associated with clinically important adverse effects to ensure that the benefits of use outweighed the risks. The most straightforward REMS programs include a medication guide and communication plan to highlight appropriate use to patients and prescribers. More complicated REMS programs for drugs with the most serious safety concerns involve Elements to Assure Safe Use (ETASU),^2^ such as required patient enrollment in a registry; person, place, and volume restrictions on dispensing; and physician and pharmacist certification.^3^

As of January 2021, 51 FDA-approved drugs or drug classes had REMS programs with ETASU.^4^ Little is known about the degree to which these programs foster safer drug use, better health outcomes, and improved patient experiences with their illnesses.^5^ A 2013 report^6^ by the Department of Health and Human Services Office of Inspector General questioned whether the FDA had adequate evidence to determine if the REMS system was achieving its goals.

Some studies of prescribing trends using insurance claims data show a variable effect of REMS programs with ETASU. Although the programs can reduce prescribing drugs for non-FDA approved indications,^7^ prescribing of covered drugs to certain populations at increased risk of adverse events can also persist.^8^ However, few analyses of REMS programs with ETASU provide insight into patient experiences and perceptions.^9,10,11^ A study by the manufacturer of the teratogenic multiple myeloma drugs lenalidomide and thalidomide found a high rate of self-reported adherence with birth control requirements (96%) and comprehension of teratogenic risk (98%).^12^ Another investigation reported that while 89% of patient educational materials from 23 REMS programs met the criteria for being understandable, only 49% were deemed actionable, resulting in the reader knowing how to act on the messages being communicated.^13^ Exploring patient and caregiver experiences with and perceptions of REMS programs with ETASU is critical for regulators and policy makers to understand the impact of these programs and to identify ways to optimize their design and implementation.

## Methods

This qualitative study followed the Consolidated Criteria for Reporting Qualitative Research (COREQ) reporting guideline and was approved by the institutional review board (IRB) at Brigham and Women’s Hospital. Informed consent was implied by voluntary participation in the interviews and facilitated by providing participants with a fact sheet detailing the goals and scope of the study, the rights of participants, and the contact information of the principal investigator and IRB.

### REMS Program Selection

To ensure the selection of programs with a meaningful impact on patient experiences, we first ranked the 40 REMS programs with ETASU available at study initiation (January 2016) based on the extent of safety requirements they included. We evaluated 4 criteria: (1) prescriber certification (score 0 if no and 1 if yes), (2) screening or follow-up monitoring (score 0 if no, 1 if recommended, 2 if mandated, and 3 if data reporting requirements), (3) dispensing restrictions (score 0 if no, 1 if pharmacy certification required and 2 if only limited specialty pharmacies could qualify), and (4) manufacturer access to patient health information (score 0 if no and 1 if yes).

We summed scores across the criteria and picked 4 FDA-approved REMS programs with ETASU scoring above the median for which indication-specific patient support groups existed that could serve as a recruitment source. The final cohort included programs for natalizumab, riociguat, sodium oxybate, and vigabatrin. Details on the indications and risks of the drugs and the features of their programs are provided in Table 1.

**Table 1.** REMS-Covered Study Drugs

Drug	Indication	Safety risk prompting REMS Program	Screening or monitoring requirement	Dispensing limitations
Natalizumab	Crohn disease, multiple sclerosis	PML, an opportunistic, often fatal infection caused by the human polyomavirus 2, the estimated incidence of which is 11 of 1000 among individuals with 3 known risk factors: (1) presence of antihuman polyomavirus 2 antibodies, (2) prior immunosuppressant use, and (3) long-term (>2 y) natalizumab use	Recommended monitoring for PML at 3 and 6 months and every 6 months thereafter; mandatory submission of discontinuation monitoring form	Certified pharmacies
Riociguat	Chronic thromboembolic pulmonary hypertension, pulmonary arterial hypertension	Birth defects, including an increased rate of cardiac ventricular-septal defect, maternal toxicity, and spontaneous abortions	Females of reproductive potential: mandatory baseline and monthly pregnancy tests	Certified pharmacies
Sodium oxybate	Cataplexy or excessive daytime sleepiness in narcolepsy	Central nervous system and respiratory depression, associated with concomitant use of sedative hypnotics or alcohol	Screening for substance abuse; mandatory monitoring at 3 months	Centralized pharmacy (mail only)
Vigabatrin	Refractory complex partial seizures (>10 y), infantile spasms (<2 y)	Mild-to-severe vision loss in 30% or more of patients, associated with increasing dose and cumulative exposure	Mandatory vision testing every 90 d^a^	Certified pharmacies

Abbreviations: PML, progressive multifocal leukoencephalopathy; REMS, risk evaluation and mitigation strategy.

^a^ Mandatory vision testing was removed as a component of the REMS program for vigabatrin in June 2016.

### Interview Guide, Patient and Caregiver Recruitment, and Conduct of Interviews

To capture patient and caregiver experiences and perspectives, we used semistructured telephone interviews. The interview questions with follow-up prompts covered education, medication access, and data sharing (eAppendix 1 in the Supplement). Patients were eligible to participate if they had taken the drug of interest within the prior 2 years. For vigabatrin, we expanded participation to caregivers of patients, while for riociguat—a known teratogen with a REMS program intended to prevent its use in pregnant women—we restricted participants to adult women of reproductive age. We recruited patients and caregivers via email solicitations to patient groups, supplemented by advertisements on social media (Twitter) and Craigslist, a classified advertisements website (in geographically diverse settings). All patients and caregivers who completed an interview were offered $50 gift cards. Each interview was conducted via phone by trained members of the research team between August 2016 and August 2017, lasted approximately 1 hour, and was recorded and transcribed without personal identifiers.

### Content Analysis

We used qualitative content analysis to summarize patient and caregiver responses to the open-ended questions.^14,15^ This method of qualitative analysis offers an approach to condense or categorize text to identify patterns systematically. Text can be summarized using deductive categories defined a priori as well as categories derived inductively based on concepts newly identified through constant comparison or by line-by-line reading of the interview text.^16^ The research team generated a consensus start list of codes for content analysis after independently reading the transcripts. One member of the team then coded each transcript, meeting with the rest of the team to review and refine the list and code definitions. A second person independently coded a subset of the transcripts to check for consistency in text interpretation and coding application, after which discrepancies were reconciled. Finally, the research team reviewed the data to identify themes within and across codes. All interviews were conducted between 2016 and 2017, with initial analysis performed in 2017 and reanalysis performed in 2021. No statistical testing was conducted.

## Results

After conducting interviews with 63 participants, we found numerous repetitive messages and concluded that we achieved thematic saturation. Among the 63 participants, 46 (73%) were female. Twenty-five patients (40%) had taken natalizumab (13 for multiple sclerosis, 10 for Crohn disease, and 2 for multiple sclerosis and Crohn disease), 10 (16%) riociguat, 15 (24%) sodium oxybate, and 10 (16%) vigabatrin. One patient had taken both natalizumab and vigabatrin, and 4 (6%) were caregivers of patients who had taken vigabatrin. Twenty-four participants (38%) were recruited through Craigslist, 23 (37%) through patient support groups, 9 (14%) through patient referrals, 4 (6%) through physician referrals, and 3 (5%) through social media. Over two-thirds of participants took the drug in question for at least 12 months (eTable in the Supplement).

After reviewing code-based extracts of the transcripts (eAppendix 2 in the Supplement), 4 primary themes emerged: (1) awareness of the REMS program and drug risks, (2) impact of the REMS program on decision-making, (3) access to the REMS-covered drug, and (4) perceptions of REMS-based data collection (Table 2).

**Table 2.** Selected Participant Comments Grouped by Primary Themes

Theme	Sample Response
**Awareness of the REMS program and drug risks**	
Awareness of REMS program	I'm not very aware of a safety program beyond that you have to get tested. I think it was before every injection I had to get tested to make sure I didn't have the [human polyomavirus 2]. But I don’t know if that was the [natalizumab] program or my doctor. (Patient taking natalizumab)
	A special safety program? I'm not 100% sure what you mean by that. I know that I have to take monthly pregnancy tests and use extra protection in my daily life. (Patient taking riociguat)
	I know that it required very strict guidelines as far as sending it out and shipping it. Being controlled as far as that's concerned. (Patient taking sodium oxybate)
	Why didn’t I know about this? (Caregiver of patient taking vigabatrin)
Awareness of drug risks	
Natalizumab	I know that for me the biggest one was the risk of PML. I think that's something that you get when if you test positive for a certain virus, you have a higher developed risk of getting that brain thing, virus that could potentially either paralyze or kill you. That was the one that I was most concerned with.
	The risks. I know I have a low immune system. It may, I’m not sure, but it may cause an infection in the brain if not being watched for it. That’s I think about it.
Riociguat	One of the primary and most dangerous risks is fetal harm. You have to not be trying to get pregnant or pregnant at the time. You also have to make sure you’ve discontinued treatment for at least a full month.
	Fetal defects. Severe fetal defects. Nausea. Rash. But the fetal defects are the big ones.
Sodium oxybate	There are psychological risks like depression and anxiety and psychosis and suicide. Also, you can stop breathing in the middle of the night especially if you use it with other drugs or alcohol.
	I know all the risks. It’s trouble breathing. Or it can accelerate. Sorry. I can’t remember the name of thing that when you sleep you can’t breathe. But it can make that worse. It can be dangerous because it knocks you out pretty much. If you’re up and about it’s not a good plan. It could be dangerous. Falling or whatever. One of the risks I know is that it has a lot of salt in it. That’s obviously a definite misuse of the drug, but it still does upset those who take it regularly. High cholesterol. I’m sure that’s not it. But that’s all of the main ones that I can think of right now.
Vigabatrin	They told me it can really affect my vision, tunnel vision, like a big effect on my vision. […] They’ve given me “Don’t get pregnant,” that whole thing. But the biggest thing was the vision, nothing other than the vision. It was the number one thing.
	The retinal deterioration is the big one. If there’s any other ones, I can’t remember what they are.
Impact of the REMS Program on decision-making	Ultimately because the risk was so low and because it was being monitored so closely, that is why I decided to take it. (Patient taking natalizumab)
	I went through the procedure blindly. I said, “Finally the doctor found out what my problem is. I'll sign my life away. Just help me.” I glanced over the paperwork and almost signed it without even reading it, which is pretty sad. But I was desperate. (Patient taking natalizumab)
	I think the safety program being available is to better safeguard my health. […] I think it made [the decision to take the drug] easier. It made me feel that there was support every step of the way. (Patient taking riociguat)
	Yes and no. It made me a little reticent about taking the drug because it just seems very involved. However, I think the upside of that is that’s a good thing. It’s a good thing for them to be so involved to ensure that I don’t accidentally get pregnant while taking the medication. (Patient taking riociguat)
	Certainly the special safety program, I guess it could have conceivably scared me off thinking “Wow. This must be bad stuff.” But it didn’t. (Patient taking sodium oxybate)
	To be perfectly honest, I really wasn’t concerned about how safe it was. I just wanted to know if it was going to work because I wanted my life back. (Patient taking sodium oxybate)
	I had already made my decision. (Patient taking vigabatrin)
	It sounded like if anything that made it a little safer because it had safety precautions in place already. I liked knowing that there was some sort of established protocol for how to have it. (Patient taking vigabatrin)
Access to the REMS-covered drug	
Natalizumab	I had a GI doctor, but she wasn't part of the program, so I couldn't get my infusions…essentially unless I switched doctors.
	Really the bad part is that I have to travel to have it done. The good part is that it’s not too frequent like an everyday injection.
Riociguat	I wasn't able to get a prescription filled. I had to sign forms. The doctor had to sign forms. I had to find a pharmacist who was a REMS-certified pharmacist…I'm going to say [it took] almost a month to make sure that this was all taken care of.
	Although somewhat burdensome, it’s a very small amount of burden.
Sodium Oxybate	They've got to have somebody here over the age of 18 to sign for it. I work, so I'm not always here. You’ve always got to make sure somebody's home. Sometimes it's not delivered on time.
	I think it’s great. […] I don’t have to take a prescription to my pharmacy. I don’t have to haggle with someone because they don’t stock it. […] It’s mailed directly to my doorstep! It’s like Amazon for medication! It’s fantastic.
Vigabatrin	When it becomes a mail order and you need it the next day or the day after, it's stressful compared to ordering at the local pharmacy and driving to it. You feel like you have less, I don't know, less control of the whole thing.
	It’s actually really easy. […] I do it at a specialty pharmacy that’s right around the block from my apartment so it’s really convenient.
Perception of REMS program data collection	I have no problem with them having the information that they need. If they need to gather information to make a better product, it’s only going to benefit me. It’s going to benefit them, too. But it’s going to benefit me. So, I’m okay with that. (Patient taking natalizumab)
	I almost felt like they basically don’t want to help you if you didn’t want to give them that information. […] They didn’t really explain why they needed the information. It said they can have it and they didn’t say what exactly they’re going to do with it or who else they’re going to share it with. […] I didn’t like that option. How long do they keep information? What if I took it? Then decided I don’t want—I want to rescind my approval of it. Stuff like that. (Patient taking natalizumab)
	I like my privacy. I like to be secure. And I don't like all these people sharing my information. I don't like it at all. But it's a requirement, and I don't have a lot to say about it. It's not my call to say “Hey, you know what? It's my information. You don't have my permission.” Because if I am not doing it, they don't give me the medication. (Patient taking riociguat)
	I don’t have a problem with a company wanting information that’s potentially going to help me. (Patient taking riociguat)
	I feel like there are people who aren’t comfortable sharing that information. It shouldn’t stop somebody from gaining the benefits of a medication because a pharmaceutical company wants more information on the demographics of the patient. (Patient taking sodium oxybate)
	It raises a red flag for me as a patient. However I can also imagine how it might be reasonable because of the nature of the drug. […] I think there should be some guidelines in place or rules in place on how that personal information can be used. Maybe how long it's retained for. (Patient taking sodium oxybate)
	I’m not overwhelmingly happy. But if there is something wrong with it or if something happens to me, I’m assuming they’re going to pick up the cost if something were to happen. (Patient taking vigabatrin)
	If you want the company to make an adjustment with the drug or at least learn quickly in real-time and get data about what's going on with patients across the country or different parts of the world that use the drug, it's definitely a great idea. I feel the good outweighs the bad. I support it. (Patient taking vigabatrin)

Abbreviations: PML, progressive multifocal leukoencephalopathy; REMS, risk evaluation and mitigation strategy.

### Awareness of the REMS Program and Drug Risks

All study participants knew that obtaining their medication required meeting specific terms, such as completing regular monitoring or using a centralized pharmacy. However, far fewer understood that these requirements were part of a special FDA-mandated program intended to promote drug safety.

A common perception held by 20 participants (32%), was that the REMS program was intended to benefit the drug manufacturer or physician. For example, a patient thought physicians were reimbursed “for referring patients” for enrollment. Other patients viewed the program as liability protection, or as one said, a way for the drug company to “cover [its back] if something down the road goes bad and then a whole bunch of people have an adverse effect maybe years later.”

Eleven participants (17%) commented that the enrollment forms associated with the REMS program were verbose, overwhelming, or vague. One patient suggested that they be simplified and shortened: “Everything all on one page, maybe.” A patient taking natalizumab wanted more understandable information about progressive multifocal leukoencephalopathy, the risk for which the REMS program existed: “They need to dumb it down for [those of] us that don’t have that medical background.”

Five participants (8%) commented that they were not sufficiently updated on new findings about their drug. For example, 2 patients taking vigabatrin said that they were poorly informed about when and why the REMS program was substantially modified in June 2016, making vision testing voluntary.^17^

### REMS Program and Decision-making

Fifty-four participants (86%) discussing their perceptions of the impact of the REMS program on their decision to take the drug had 1 of 2 responses: either it had no effect, or it made them feel more comfortable about their choice.

Participants who reported no effect gave a variety of reasons. Some reported that they had agreed to take the drug before seeing the enrollment forms. Others stated they had already been well informed about safety issues by their physician. Some said they were so motivated to find a treatment that they were willing to discount the risks.

The other major group of participants were pleased to have the REMS program in place. Some reported that it helped them “feel a bit more comfortable” or have “stable, secure peace of mind.” Two REMS programs required laboratory monitoring, oversight that was particularly important for several patients. For example, a patient taking natalizumab explained he did not know whether the FDA or the manufacturer was doing the monitoring, but he was pleased the monitoring system was in place.

Fifteen participants (24%) found the REMS program requirements excessive. Some commented that the REMS program overemphasized medication risks since all drugs have risks. As a patient taking riociguat said: “I’m all for safety. I’m just not for overkill.” Another patient taking riociguat worried how the REMS requirements would affect willingness to initiate treatment: “But I could see how a new person would be like ‘Wow. I have to sign all this information just to start taking a pill every day?’”

### Access to the REMS-Covered Drug

Thirty-four participants (54%) reported burdens accessing REMS-covered drugs unrelated to insurance coverage at some point during their course of treatment, ranging from additional but not prohibitive challenges to major obstacles. Patients taking natalizumab pointed out that not all infusion centers were enrolled in the REMS program, requiring travel to the nearest center, which may not have been close to public transportation or within reach of patient transport services. One patient noted that such problems led him to consider alternative treatments: “I have several times asked my doctor about the idea of switching me to oral medication” because of the challenge of getting to a REMS program-certified infusion center.

Some patients taking riociguat reported filling prescriptions was somewhat burdensome. Most commented on the challenge posed by visiting physicians’ offices once a month for the required pregnancy test, instead of taking the test from home. Some felt it was unnecessary for pharmacists to know the results of pregnancy testing.

The REMS program provision for sodium oxybate requiring the patient to accept and sign for the delivery of the drug to reduce the risk of diversion was widely viewed as inconvenient. One patient noted: “I would say it’s maybe a little more difficult only because you can’t just drive to your pharmacy and pick it up.”

The vision testing required for vigabatrin was generally not considered onerous. However, some participants reported challenges with finding a participating pharmacy or being home to accept a delivery. As a mother of a young patient described: “It’s just extra steps, phone calls, and additional worry that it’s going to get here on time, so she doesn’t miss a dose.”

### Perception of REMS-Based Data Collection

Some REMS programs in the study had enrollment agreements that authorized broad disclosure of personal health information to the drug manufacturer. For example, the enrollment agreement for the REMS program for vigabatrin stated, “I authorize my health care providers and health plans to disclose personal and medical information related to my use or my potential use of [vigabatrin] to the manufacturer…and its agents and contractors” to, among other things, “communicate with [the patient’s] health care providers and health plans about [the patient’s] benefit and coverage status and my medical care.”^18^

Thirty-six participants (57%) were concerned about such disclosure. One patient noted that she did not understand why the manufacturer needed her information: “I feel they just want more of my information that’s not necessarily related to [the drug].” Another patient was concerned the information would be used “for marketing purposes.” Inadequate data protection was a major worry: “Information that is disseminated or passed on can be also leaked.” Many believed that it was not fair to require them to provide personal information to a drug manufacturer to receive a prescription.

These participants wanted information deidentified and limited to the condition being treated by the drug as much as possible. Most also felt that companies should not be permitted to retain patient information for an extended period.

A positive perspective on data sharing came from 16 (25%) of participants, who believed they were trying to help future patients. One patient remarked: “I think if it could help somebody, that’s how I think we’re going to get further to maybe finding a cure or an end or something for [multiple sclerosis].” Another believed that sharing personal information could help provide insight into how the drug works among minority populations. One said that the drug manufacturer should have long-term access to her medical records: “I think it’s great just because the drug truly has been so life-changing for me. If any of the information they get from me could be helpful to somebody else or it’s a study that they’re doing, I am all for sharing that information.”

Eleven participants (17%) expressed the belief that they might benefit personally from such sharing because they expected the manufacturer to monitor them to be sure that they were getting the treatment they needed—to “see if it is working out for me” and perhaps “customize a solution” that would lead to “resolving the side-effects”—even though no manufacturers engaged in such outreach. One patient inaccurately believed that by sharing her information, the manufacturer would compensate her if she experienced an adverse event.

### Cross-Cutting Themes

We identified 3 cross-cutting themes from the primary themes: skepticism, powerlessness, and knowledge deficits. Some participants were skeptical of the risk of the REMS-covered drug, the intended beneficiaries of the REMS program, or the purpose of data collection under it. Many participants felt at the mercy of the REMS requirements because they had to comply with testing procedures, had no convenient way to receive the drug, or were obligated to share personal health information with the manufacturer. As a patient taking riociguat noted: "We're basically told what we have to do, and well, that's that." Finally, some participants lacked knowledge regarding the existence of, changes to, or findings from the program.

## Discussion

The purpose of REMS programs is to ensure that when drugs have serious side effects, the benefits of use still outweigh the risks. We found that some patients and their caregivers perceived REMS programs with ETASU to increase comfort initiating treatment. Concerns were raised about the intended beneficiaries of the programs, the quality of educational materials, the ease of accessing medications, and data privacy.

REMS programs with ETASU have benefits beyond their primary public health aims. For example, oversight requirements, like laboratory monitoring, made some patients more comfortable with initiating treatment. It is axiomatic that patients should be informed of the benefits and risks of a drug before they initiate treatment. Since REMS–covered drugs carry important risks, the comfort that these oversight requirements create may help ensure that patients adhere to their treatment plans and get the chance to experience the clinical benefits that these drugs offer. Epidemiologic studies of medication adherence can help provide insight into whether such perceptions translate into improved adherence and patient outcomes.^5^

Our study also showed how REMS programs with ETASU can be improved by creating more understandable and informative educational literature for patients. One way to do that would be to use validated educational tools to update REMS program materials. For example, a study^19^ that assessed these documents used the Patient Education Materials Assessment Tool (PEMAT), a validated instrument consisting of 24 items spanning understandability (absorbing what the text says) and actionability (grasping recommended behaviors). Using such tools would ensure that REMS materials enable patients to understand the benefits and risks of the drug, the steps required of them to minimize risks, and the primary purpose of data collection. To facilitate informed decision-making, the FDA can require that clinicians attest that they have gone over REMS educational materials and enrollment forms with patients in person.

In particular, it will be important to correct fallacies in patients’ understanding of REMS programs, the most common being that the FDA or manufacturer would be individually monitoring them when the primary aim of the programs is to ensure that the benefits of a drug’s use outweigh its risks in the aggregate. Some monitoring aspects of REMS programs with ETASU directly benefit patients while fulfilling this primary aim, such as laboratory testing before each dispensing to ensure that patients do not have heightened risk factors. However, other monitoring requirements provide benefits only at the population level, such as data sharing of personal health information with the manufacturer. It is not the case, for example, that the FDA or the manufacturer will customize a solution based on collection of these data. This REMS misconception bears some similarities to therapeutic misconception, which occurs when participants in certain clinical trials mistakenly believe that they will directly benefit from their participation.^20^ Given the importance of dispelling the REMS misconception that we observed, education should be accomplished at the initiation of the drug as well as at regular intervals during a patient’s treatment course.

Actions can also be taken to promote equitable access to drugs covered by REMS programs with ETASU. Not all patients are within a reasonable distance of certified physicians, pharmacies, or infusion centers, and for some, this distance may be a barrier to treatment. For relevant REMS programs with ETASU, the FDA can require manufacturers to routinely report the number and location of certified providers and should require more active provider recruitment in underserved areas. Additionally, flexibility can be enhanced for mail-order drugs, including patient- or caregiver-driven time and location scheduling of deliveries.

The FDA should also issue guidance on the types of personal health information that can be collected under REMS programs with ETASU, for what purposes, and over what time. Collected data should not be used for marketing purposes, which may be aided by time limits on personal health information retention. To further protect against the possible misuse of such information, the FDA can require independent administration of REMS programs with ETASU by third parties,^21^ which already operate many programs.^22^

### Limitations

Among the limitations of our study is that the sample included only 4 programs. However, the programs shared common elements with many other REMS programs with ETASU. Additionally, the patients we interviewed might not have been representative of all patients taking the 4 drugs. Finally, the percent of respondents reported as providing a particular answer did not capture differences in the strength of their responses.

## Conclusions

These findings suggest that REMS programs with ETASU may help improve comfort with initiating covered drugs, but manufacturers need to take steps to improve informed consent, maximize medication access, and bolster privacy protections. Patient and caregiver experiences can be enhanced if the FDA and manufacturers improve understanding of and trust in these important programs.

## Notes

Supplement. **eAppendix 1.** Patient Interview Guide **eAppendix 2.** Codes **eTable.** Participant Demographics
