# Supplementary material for: Patient and Caregiver Experiences With and Perceptions of Risk Evaluation and Mitigation Strategy Programs With Elements to Assure Safe Use
Source: JAMA Netw Open. 2022 Jan 20;5(1):e2144386. doi: 10.1001/jamanetworkopen.2021.44386 (PMC8777595; doi:10.1001/jamanetworkopen.2021.44386)
Supplement: Supplement. — eAppendix 1. Patient Interview Guide eAppendix 2. Codes eTable. Participant Demographics [file jamanetwopen-e2144386-s001.pdf]

Supplemental Online Content

Sarpatwari A, Brown BL, McGraw SA, et al. Patient and caregiver experiences with and perceptions of Risk Evaluation and Mitigation Strategy programs with elements to assure safe use. *JAMA Netw Open*. 2022;5(1):e2144386. doi:10.1001/jamanetworkopen.2021.44386

eAppendix 1. Patient Interview Guide
eAppendix 2. Codes
eTable. Participant Demographics

This supplemental material has been provided by the authors to give readers additional information about their work.

© 2022 Sarpatwari P et al. *JAMA Network Open.*

eAppendix 1: Patient Interview Guide

I. INTRODUCTION/BACKGROUND (10 minutes)
Good [morning/afternoon/evening]. May I speak with [patient's name]? Hi, my name is [your name], and I am calling as part of a study on a medication safety program by researchers at Harvard Medical School and Brigham and Women's Hospital in which you agreed to take part. If you complete this interview, we will send you or a charity of your choice $50 for your time and effort. Is this still a good time for us to talk?

IF NO: ARRANGE A TIME TO CALL BACK.

To begin, let me confirm that you received at least one prescription for [drug name] in the past year. Is that right?

IF NO: THANK POTENTIAL RESPONDENT FOR (HIS/HER) TIME AND END THE INTERVIEW. IF YES, CONTINUE.

How long (did you take/have you been taking) [drug name]?

IF NO LONGER TAKING: Approximately when was this?

How frequent (were/are) your (refills/infusions)?

Thanks. To clarify, I am not a medical doctor and am not able to offer medical advice, so if any questions come up about your own treatment plans, you should discuss them with your physician. If there is a question that you would rather not answer, we can skip it. This interview is being recorded so that our team can accurately capture your responses, which we hope to use to determine how the special safety program can be improved. Let's begin.

II. EDUCATION (30 minutes)
<u>Background understanding</u>

1. As far as you know, what risks are associated with taking [drug name]?

2. How did you learn about risks associated with taking [drug name]?

3. Thinking about risks associated with taking [drug name], is there anything more you wish you (knew/had known)? If so, what?

4. Thinking back to your very first conversation seeking treatment for your condition using [drug], how long was the process to start taking the drug? How many physicians were involved in this process?

<u>Questions about safety program</u>

5. **IF SAFETY PROGRAM NOT MENTIONED** IN RESPONSE TO FIRST SEQUENCE: Are you aware that [drug name] requires a special safety program?

© 2022 Sarpatwari P et al. *JAMA Network Open.*

a. **IF NO**: This safety program is called risk evaluation and mitigation strategies or REMS and is aimed at ensuring a drug with safety concerns is used in a way that its benefits outweigh its risks. Do you think knowing about the safety program would have impacted your willingness to take the drug? Why or why not?

b. **IF YES**: Did knowledge of the safety program impact your willingness to take the drug? Why or why not?

6. **IF SAFETY PROGRAM MENTIONED** IN RESPONSE TO FIRST SEQUENCE: Did knowledge of the safety program for [drug name] impact your willingness to take the drug? Why or why not?

7. How do you view the risk-benefit tradeoff in taking [drug name]?

8. Do you recall signing an enrollment/agreement form before starting your treatment with [drug name]?

<u>IF NO TO QUESTION 8, PROCEED TO QUESTION 13</u>

<u>IF YES TO QUESTION 8, ASK QUESTIONS 9-12</u>

9. Can you recall if there were specific terms that you agreed to follow?

<u>IF YES TO QUESTION 9</u>

a. What were they?

<u>PROVIDE INFORMATION ON REMS</u>

10. What do you think about the enrollment/agreement form? (PROBE IF NOT MENTIONED: Do you find the enrollment/agreement form helpful? Are the terms necessary? invasive? costly?)

    a. Did the enrollment/agreement form affect how you think about your treatment? (PROBE IF NOT MENTIONED: To what degree, if at all, do you think it impacted your perception of the risk of [drug name]?)

11. Was your physician also required to sign the enrollment/agreement form?

    a. **IF YES**: How did you feel about that?

    b. **IF NO**: If you knew that your physician was required to sign an enrollment/agreement form, would that have changed how you felt about taking [drug name]. If so, how?

12. (Did you do/have you done) anything differently as a result of signing the enrollment/agreement form? If so, what?

<u>IF QUESTIONS 8-12 ASKED, PROCEED TO QUESTION 15</u>
<u>PROVIDE INFORMATION ON REMS</u>

© 2022 Sarpatwari P et al. *JAMA Network Open.*

13. What do you think about being asked to agree to those terms?

14. Does requiring your physician to sign the agreement with you make a difference? Why or why not?
15. As part of the safety program, were you asked to answer questions or take a quiz to test your knowledge?

<u>IF NO TO QUESTION 15, ASK QUESTIONS 16-17</u>

16. Would you have wanted such questions or test? Why or why not?

17. What do you see as the advantages and disadvantages of such a requirement?

<u>IF YES TO QUESTION 15, ASK QUESTIONS 18-19</u>

18. What did you think about these questions? (PROBE IF NOT MENTIONED: Were the questions helpful or not? necessary?)

19. To what extent, if any, did questions affect how you think about the risks associated with taking the drug?

III. MEDICATION ACCESS (10 minutes)
Next, I'd like to ask you about the filling your prescription for [drug name] and about difficulties, if any, you have encountered with the drug.

20. How do you typically get your medications?

21. How do you get [drug name]?
    (PROBE AS NEEDED: Do you use a specialty pharmacy? Do you go to an infusion center or a hospital setting to take your medication?)

22. How has your experience been getting [drug name]? Has it been easier, more difficult, or about the same compared to other medications? In what way?
    a. (Did you have/have you had) issues with your insurance? If so, what?
    b. (Did/does) your doctor require information from you between prescriptions? If so, what?

23. (Did you experience/have you experienced) any difficulty getting questions about [drug name] answered? If yes, please explain.

IV. TRUST (10 minutes)
Now, I'd like to discuss the issue of health privacy.

24. You answered that you [were/were not] required to submit enrollment/agreement forms to the drug company. Do you know if your physician or pharmacist is required to provide other information to the drug company as part of the safety program?

© 2022 Sarpatwari P et al. *JAMA Network Open.*

25. **IF ACCESS TO PERSONAL HEALTH INFORMATION REQUIRED**: The terms of the safety program state [provision]. What do you think about this provision? (PROBE IF NOT MENTIONED: Are there any conditions you would like to see imposed on such access? If so, what?)
    a. How much information is appropriate to provide? How often should it be provided? For what purposes can it be used?

26. **IF ACCESS TO PERSONAL HEALTH INFORMATION NOT REQUIRED**: Some safety programs require drug company access to personal health information. Are there any conditions you would like to see imposed on such access? If so, what?
    a. How much information is appropriate to provide? How often should it be provided? For what purposes can it be used?

27. Did you experience any adverse events? Can you describe any consequent interactions with your physician and/or the drug company?

28. Given the insights you've shared, how, if at all do you think the drug safety system can be improved?

We will be in touch soon regarding compensation. Would you like to provide the address that we can use to send your gift card now or do you prefer to follow up with this information via email?

If additional questions arise, please contact the study principal investigator, Dr. Ameet Sarpatwari at 617-525-8890.

Thank you for your time and your insights. I appreciate your speaking with me.

© 2022 Sarpatwari P et al. *JAMA Network Open.*

eAppendix 2. Codes

1. Code Family: Drug Experiences and Choices
    a. History
    b. Risk/Benefit
    c. Adverse Events

2. Code Family: REMS and Perceptions about Treatment
    a. Information on Drug and Risks
    b. Risk Knowledge
    c. Impact of REMS on Perception

3. Code Family: REMS and Access to Medications
    a. Access to Medication
    b. Insurance and Cost

4. Code Family: Awareness of REMS Elements
    a. Awareness of REMS
    b. Enrollment MD
    c. Enrollment Patients

5. Code Family: Data
    a. Autonomy
    b. Pharma and Data Collection

6. Code Family: Value of REMS
    a. Improvements to REMS
    b. Quizzes and Assessing Patient Knowledge
    c. Utility

© 2022 Sarpatwari P et al. *JAMA Network Open.*

eTable. Participant Demographics

Patient No.	Drug	Sex	Recruitment method	Time on drug, mo
1	Riociguat (Adempas)	Female	Patient support group	36
2	Riociguat (Adempas)	Female	Craigslist	24
3	Riociguat (Adempas)	Female	Physician referral	12
4	Riociguat (Adempas)	Female	Patient referral	24
5	Riociguat (Adempas)	Female	Patient referral	12
6	Riociguat (Adempas)	Female	Patient referral	6
7	Riociguat (Adempas)	Female	Patient referral	6
8	Riociguat (Adempas)	Female	Patient referral	6
9	Riociguat (Adempas)	Female	Patient referral	6
10	Riociguat (Adempas)	Female	Patient support group	24
11	Vigabatrin (Sabril)	Female	Patient support group	156
12	Vigabatrin (Sabril)	Female	Craigslist	18
13	Vigabatrin (Sabril), and natalizumab (Tysabri)-MS	Female	Craigslist	24
14	Vigabatrin (Sabril)	Female	Craigslist	12
15	Vigabatrin (Sabril)	Female	Craigslist	36
16	Vigabatrin (Sabril)	Male	Craigslist	36
17	Vigabatrin (Sabril)	Female	Craigslist	36
18	Vigabatrin (Sabril)	Female	Twitter	72
19	Vigabatrin (Sabril)	Male	Craigslist	Unknown
20	Vigabatrin (Sabril)	Female	Craigslist	8
21	Vigabatrin (Sabril)	Female	Craigslist	18
22	Vigabatrin (Sabril)	Female	Patient referral	18
23	Vigabatrin (Sabril)	Male	Craigslist	24
24	Vigabatrin (Sabril)	Male	Craigslist	12
25	Natalizumab (Tysabri)-Crohn’s	Female	Patient support group	3
26	Natalizumab (Tysabri)-Crohn’s	Female	Patient support group	72
27	Natalizumab (Tysabri)-Crohn’s	Female	Patient support group	60
28	Natalizumab (Tysabri)-Crohn’s	Female	Patient support group	84
29	Natalizumab (Tysabri)-Crohn’s and MS	Female	Twitter	96
30	Natalizumab (Tysabri)-Crohn’s and MS	Male	Craigslist	12

© 2022 Sarpatwari P et al. *JAMA Network Open.*

31	Natalizumab (Tysabri)-Crohn's	Male	Craigslist	72
32	Natalizumab (Tysabri)-Crohn's	Female	Craigslist	12
33	Natalizumab (Tysabri)-Crohn's	Unknown	Craigslist	18
34	Natalizumab (Tysabri)-Crohn's	Male	Craigslist	60
35	Natalizumab (Tysabri)-Crohn's	Male	Craigslist	12
36	Natalizumab (Tysabri)-Crohn's	Female	Craigslist	18
37	Natalizumab (Tysabri)-MS	Male	Physician referral	72
38	Natalizumab (Tysabri)-MS	Male	Patient support group	24
39	Natalizumab (Tysabri)-MS	Female	Craigslist	84
40	Natalizumab (Tysabri)-MS	Female	Craigslist	12
41	Natalizumab (Tysabri)-MS	Female	Craigslist	36
42	Natalizumab (Tysabri)-MS	Female	Twitter	72
43	Natalizumab (Tysabri)-MS	Female	Physician referral	72
44	Natalizumab (Tysabri)-MS	Female	Patient referral	12
45	Natalizumab (Tysabri)-MS	Female	Physician referral	36
46	Natalizumab (Tysabri)-MS	Male	Craigslist	12
47	Natalizumab (Tysabri)-MS	Female	Craigslist	84
48	Natalizumab (Tysabri)-MS	Female	Patient referral	13
49	Sodium oxybate (Xyrem)	Female	Patient support group	4
50	Sodium oxybate (Xyrem)	Female	Patient support group	18
51	Sodium oxybate (Xyrem)	Male	Patient support group	24
52	Sodium oxybate (Xyrem)	Female	Patient support group	60
53	Sodium oxybate (Xyrem)	Female	Patient support group	36
54	Sodium oxybate (Xyrem)	Male	Patient support group	94
55	Sodium oxybate (Xyrem)	Female	Patient support group	Unknown
56	Sodium oxybate (Xyrem)	Female	Patient support group	180
57	Sodium oxybate (Xyrem)	Female	Patient support group	144
58	Sodium oxybate (Xyrem)	Female	Patient support group	24
59	Sodium oxybate (Xyrem)	Female	Patient support group	12
60	Sodium oxybate (Xyrem)	Female	Patient support group	72

© 2022 Sarpatwari P et al. *JAMA Network Open.*

61	Sodium oxybate (Xyrem)	Male	Patient support group	42
62	Sodium oxybate (Xyrem)	Male	Patient support group	120
63	Sodium oxybate (Xyrem)	Male	Patient support group	36

Abbreviation: MS, multiple sclerosis.

© 2022 Sarpatwari P et al. *JAMA Network Open.*
